# Supplementary material for: Six-month psychopathological symptom trajectories following the COVID-19 outbreak: Contrasting mental health outcomes between nurses and the general population
Source: PLoS One. 2024 Apr 16;19(4):e0301527. doi: 10.1371/journal.pone.0301527 (PMC11020497; doi:10.1371/journal.pone.0301527)
Supplement: S1 Checklist — (DOCX) [file pone.0301527.s001.docx]

STROBE Statement—checklist of items that should be included in reports of observational studies

|  | Item No. | Recommendation | Page  No. | Relevant text from manuscript |
| --- | --- | --- | --- | --- |
| **Title and abstract** | 1 | (*a*) Indicate the study’s design with a commonly used term in the title or the abstract | 2 | The objective of the current study is to conduct a longitudinal evaluation |
|  |  | (*b*) Provide in the abstract an informative and balanced summary of what was done and what was found | 2 | Self-report questionnaires were administered online to a sample of 180 nurses and 158 individuals from the general population for the baseline assessment (T1) and follow-up at 6 months (T2). (…) Levels of fear of COVID-19 declined significantly from T1 to T2 in both groups. |
| Introduction | | | |  |
| Background/rationale | 2 | Explain the scientific background and rationale for the investigation being reported | 5-6 | The results from longitudinal studies are still not consistent about the trajectory of symptoms. (…) Contrasting mental health outcomes between samples enables the development of evidence-based guidelines on how to respond to this and future pandemics or infection waves and tailor prevention and intervention according to the needs of specific at-risk groups. |
| Objectives | 3 | State specific objectives, including any prespecified hypotheses | 7 | The main goal of the current study was to longitudinally assess the levels of depressive, anxiety, trauma and fear of COVID-19 symptoms in nurses and the general population over a six-month period. |
| Methods | | | |  |
| Study design | 4 | Present key elements of study design early in the paper | 7 | It involves a retrospective, longitudinal design with two assessment timepoints: baseline (T1) and follow-up at 6 months (T2). |
| Setting | 5 | Describe the setting, locations, and relevant dates, including periods of recruitment, exposure, follow-up, and data collection | 8 | The baseline assessment (T1) was conducted between September 2021 and May 2022, while the second point of data collection (T2) was performed between May 2022 and December 2022. The first step of the survey was to inform the participants about the study aims and procedures, as well as the voluntary, anonymous, and confidential nature of the investigation. (…)They completed the Portuguese validated versions of the self-report instruments, using a web-based platform (LimeSurvey®). |
| Participants | 6 | (*a*) *Cohort study*—Give the eligibility criteria, and the sources and methods of selection of participants. Describe methods of follow-up  *Case-control study*—Give the eligibility criteria, and the sources and methods of case ascertainment and control selection. Give the rationale for the choice of cases and controls  *Cross-sectional study*—Give the eligibility criteria, and the sources and methods of selection of participants | 8 | The research sample was invited to participate in this study through social and traditional media platforms and institutional email (…) The two inclusion criteria for the community sample to participate in this study were: being older than 18; and being able to understand Portuguese and living in Portugal. To answer the survey specific for the group of nurses, there were a single inclusion criterion: being a nurse working in a Portuguese hospital or any other health care institution. |
|  |  | (*b*) *Cohort study*—For matched studies, give matching criteria and number of exposed and unexposed  *Case-control study*—For matched studies, give matching criteria and the number of controls per case |  |  |
| Variables | 7 | Clearly define all outcomes, exposures, predictors, potential confounders, and effect modifiers. Give diagnostic criteria, if applicable | 9-11 | Subection “Measures” |
| Data sources/ measurement | 8* | For each variable of interest, give sources of data and details of methods of assessment (measurement). Describe comparability of assessment methods if there is more than one group | 9-11 | Subection “Measures” |
| Bias | 9 | Describe any efforts to address potential sources of bias | 8 | (…) forced answering (i.e., forcing respondents to answer each question to proceed through the questionnaire) was used (…) |
| Study size | 10 | Explain how the study size was arrived at | 8 | Respondents who consented to provide an e-mail contact received an invitation to voluntarily participate again in the study at the 6-month follow-up. |

Continued on next page

| Quantitative variables | 11 | Explain how quantitative variables were handled in the analyses. If applicable, describe which groupings were chosen and why | 11 | Statistical Package for the Social Sciences (SPSS, version 27.0; IBM SPSS, Chicago, IL, USA) was the software selected to perform data analyses |
| --- | --- | --- | --- | --- |
| Statistical methods | 12 | (*a*) Describe all statistical methods, including those used to control for confounding | 11-13 | Section “Data Analysis” |
|  |  | (*b*) Describe any methods used to examine subgroups and interactions | 12 | Descriptive statistics were obtained for all variables under study, and differences in sociodemographic and clinical variables were tested through mean differences tests (Student’s t tests) or frequency differences for categorical variables (chi-square tests). |
|  |  | (*c*) Explain how missing data were addressed | 8 | (…) forced answering (i.e., forcing respondents to answer each question to proceed through the questionnaire) was used (…) |
|  |  | (*d*) *Cohort study*—If applicable, explain how loss to follow-up was addressed  *Case-control study*—If applicable, explain how matching of cases and controls was addressed  *Cross-sectional study*—If applicable, describe analytical methods taking account of sampling strategy | 12 | A mixed model ANOVA was used to assess the effects of group and time on individuals’ mental health outcomes in the aftermath of the COVID-19 pandemic (depressive, anxiety, and trauma symptoms and fear of COVID-19). |
|  |  | (*e*) Describe any sensitivity analyses | 13 | For the comparison analyses, effect-size measures (partial eta squared) were presented considering $\eta_{P}^{2}=$0.01 as a small effect, $\eta_{P}^{2}=$0.06 as a medium effect and $\eta_{P}^{2}=$0.14 as a large effect [48]. |
| Results | | | | |
| Participants | 13* | (a) Report numbers of individuals at each stage of study—eg numbers potentially eligible, examined for eligibility, confirmed eligible, included in the study, completing follow-up, and analysed | 8 | Of the 1335 participants (672 nurses and 663 individuals from the general population) on the baseline assessment, 338 completed the follow-up (25.3%), with a mean age of 38.4 (SD = 10.58), 89.3% (n = 302) women, 10.1% (n = 34) men, and 0.2% (n = 2) nonbinary individuals. |
|  |  | (b) Give reasons for non-participation at each stage | 7 | Given the abundant participation requests in postdisaster research, low response rates have been frequently indicated as a major limitation of longitudinal studies in the context of the COVID-19 pandemic |
|  |  | (c) Consider use of a flow diagram | 14 | Table 1 |
| Descriptive data | 14* | (a) Give characteristics of study participants (eg demographic, clinical, social) and information on exposures and potential confounders | 13 | Subsection “Sociodemographic and clinical characteristics of the sample” |
|  |  | (b) Indicate number of participants with missing data for each variable of interest |  |  |
|  |  | (c) *Cohort study*—Summarise follow-up time (eg, average and total amount) |  |  |
| Outcome data | 15* | *Cohort study*—Report numbers of outcome events or summary measures over time |  |  |
|  |  | *Case-control study—*Report numbers in each exposure category, or summary measures of exposure |  |  |
|  |  | *Cross-sectional study—*Report numbers of outcome events or summary measures | 18 | Table 4 |
| Main results | 16 | (*a*) Give unadjusted estimates and, if applicable, confounder-adjusted estimates and their precision (eg, 95% confidence interval). Make clear which confounders were adjusted for and why they were included |  | Subsection “Comparison of depressive, anxiety, trauma, and fear of COVID-19 between nurses and the general population over time” |
|  |  | (*b*) Report category boundaries when continuous variables were categorized | 15 | Table 2 |
|  |  | (*c*) If relevant, consider translating estimates of relative risk into absolute risk for a meaningful time period |  |  |

Continued on next page

| Other analyses | 17 | Report other analyses done—eg analyses of subgroups and interactions, and sensitivity analyses | 19 | Table 5 |
| --- | --- | --- | --- | --- |
| Discussion | | | | |
| Key results | 18 | Summarise key results with reference to study objectives | 20 | This study aimed to explore the differences in the longitudinal evolution of mental health outcomes between nurses and the general population over a six-month period. The main findings may be summarized as follows: first (…). |
| Limitations | 19 | Discuss limitations of the study, taking into account sources of potential bias or imprecision. Discuss both direction and magnitude of any potential bias | 23 | Subsection “Limitations and Future Directions” |
| Interpretation | 20 | Give a cautious overall interpretation of results considering objectives, limitations, multiplicity of analyses, results from similar studies, and other relevant evidence | 23-24 | Subsection “Public health relevance” |
| Generalisability | 21 | Discuss the generalisability (external validity) of the study results | 23 | Finally, the specific country and period of the pandemic in which data were collected may limit the generalizability of findings to other cultural contexts and moments of this ever-changing health crisis (in terms of positive COVID-19 cases and associated lockdown measures), thus reinforcing the need to replicate this research for different populations and stages of the pandemic. |
| Other information | |  | | |
| Funding | 22 | Give the source of funding and the role of the funders for the present study and, if applicable, for the original study on which the present article is based | 30 | This study was supported by the Center for Research in Neuropsychology and Cognitive–Behavioral Intervention (CINEICC) at the University of Coimbra (UIDB/PSI/00730/2020) and by a doctoral grant (2020.07981.BD) awarded to the first author (Portuguese Foundation for Science and Technology/MCTES). The APC was funded by the Center for Research in Neuropsychology and Cognitive-Behavioral Intervention (CINEICC) at the University of Coimbra. |

*Give information separately for cases and controls in case-control studies and, if applicable, for exposed and unexposed groups in cohort and cross-sectional studies.

**Note:** An Explanation and Elaboration article discusses each checklist item and gives methodological background and published examples of transparent reporting. The STROBE checklist is best used in conjunction with this article (freely available on the Web sites of PLoS Medicine at http://www.plosmedicine.org/, Annals of Internal Medicine at http://www.annals.org/, and Epidemiology at http://www.epidem.com/). Information on the STROBE Initiative is available at www.strobe-statement.org.
