# Supplementary material for: Six-month psychopathological symptom trajectories following the COVID-19 outbreak: Contrasting mental health outcomes between nurses and the general population
Source: PLoS One. 2024 Apr 16;19(4):e0301527. doi: 10.1371/journal.pone.0301527 (PMC11020497; doi:10.1371/journal.pone.0301527)
Supplement: Attachment — Submitted filename: Response Letter_Longitudinal_PLOS.docx [file pone.0301527.s002.docx]

Catarina Vitorino

Center for Research in Neuropsychology and Cognitive-Behavioral Intervention

Faculty of Psychology and Educational Sciences, University of Coimbra

Rua do Colégio Novo

3000-115 Coimbra, Portugal

The Editor-in-Chief of

*PLOS one*

Prof. Dr. Emily Chenette

Coimbra, 1^st^ March 2024

*Subject:* Submission of a revised manuscript to *PLOS one.*

Dear Prof. Dr. Emily Chenette:

Please find attached the revised manuscript for the original research article “Six-month psychopathological symptom trajectories following the COVID-19 outbreak: Contrasting mental health outcomes between nurses and the general population”, which we are most pleased to submit to “PLOS one”.

The attached manuscript was carefully revised in agreement with the review (please see below the detailed response to each one of the reviewers’ comments), as well as with the PLOS one’s style requirements. The reference list was also revised and amended according to “Vancouver” style.

Regarding data availability, we will ensure long-term storage and access by giving all relevant data and reported findings to other members of our Research Center who did not collaborate with the present study. As such, external data requests may be sent to the Center for Research in Neuropsychology and Cognitive-Behavioral Intervention by email ([cineicc@fpce.uc.pt](mailto:cineicc@fpce.uc.pt)), in case the corresponding author becomes unavailable.

We would like to extend our special thanks to the reviewers for their constructive feedback on our manuscript with such insightful recognition: *The authors did a good review of previous literature to support their research. [Reviewer 2].*

Thank you very much for allowing the resubmission of the manuscript, as it is a great honour to have the opportunity to improve our work.

We appreciate the attention given to our research and will be certainly looking forward to your reply.

With our respectful compliments,

(Catarina Alves Vitorino, corresponding author)

**Reviewer #1**

This is a 6-month longitudinal cohort study comparing the questionnaire responses of a sample of 180 nurses to those from a sample of 158 persons in the general population during the Covid pandemic. Questions covered issues of depressive, anxiety, trauma and fear of Covid-19 symptoms. The objective is to evaluate the stability or change in adaptation outcomes between these groups at two time points separated by 6 months.

**Comment**

It would be good to know the power of the study as it would add to the validity of the study.

The attrition rate of 75% between T1 and T2 is high and this would affect the validity of the study and introduce bias into the study. So have there been attempts to use the "intention to treat" protocol to account for the attrition and has this been planned for in the planning phase of the study?

- *Thank you for raising this point. We do endorse your concern. The influence of the attrition rate on the results is depicted in the Limitations section, especially due to “research fatigue” in the aftermath of a mass traumatic event such as the COVID-19 pandemic (Patel et al. 2020). However, the literature suggests that the intention-to-treat protocol usually applies to randomized clinical trials (Fisher et al, 1990). According to your note, we tried to clarify this topic* (“*However, the literature shows that estimates of associations between variables do not seem to be affected by attrition rate when it is dependent on follow-up variables, which may support the generalizability of the present study. Nevertheless, future studies should address this issue by using more sophisticated analysis to handle missing values and reduce bias (e.g., multiple imputation analysis) (Gustavson et al., 2012).”).*

On data presentation, data presented in the tables need not be repeated in the text but significant points should be highlighted in the text.

- *As you recommended, information presented in tables were better described in the text (e.g., “Regarding the clinical levels of symptoms presented in Table 2, nurses reported higher levels than the general population for all measures, ranging between 22.8% and 31.7% at T1, and 21.7% and 43.3% at T2. Among the general population, symptoms with clinical significance ranged between 15.8% and 29.1% at T1, 11.4% and 24.7% at T2.”)*

You have highlighted some of the biases and shortcomings of the study.

- *Thank you for acknowledging our best efforts to provide to the PloS One‘s readers with a clear, and reliable research outcome.*

In Fig 1, the slopes of the lines for depression, anxiety and trauma were divergent between the nursing group and the general population. This suggests that over time, the symptoms continued to rise in the nursing group in contrast to the general population where the symptoms tended to decline over time, between T1 and T2. Fear of the pandemic tended to decline over time for both groups.

- *Thank you for sharing your insights on the analysis of these results, which are very much aligned with the comments that we have elaborated in the Discussion section: “The main findings may be summarized as follows: first, nurses generally reported higher levels of symptoms than the general population; second, the mental health outcomes of nurses tended to deteriorate over time, except for fear of COVID-19; third, all mental health outcomes of the general population tended to improve from T1 to T2; fourth, the difference in depressive, anxiety, and trauma symptoms over time was significant between nurses and the general population; and finally, fear of COVID-19 significantly decreased over time for both groups, but especially for the general population.”.*

**Reviewer #2**

The manuscript entitled "Six-month psychopathological symptom trajectories following the COVID-19 outbreak: Contrasting mental health outcomes between nurses and the general population” is well organized and written clearly.

The main goals of the paper are clear, and the research is relevant to the field of health psychology, specifically to the study of the impact of COVID-19 outbreak on health professionals. The research has a longitudinal design, which makes the results more valid and meaningful.

The abstract is well structured and summarizes the methodology and results of the study.

The authors did a good review of previous literature to support their research.

Regarding the Method, the description of the characteristics of the general population sample could be enriched so that it was clearer who the participants were (e.g., professional status or job), also because sample was collected through online recruitment.

- *Thank you so much for your careful and comprehensive review. The general population was better described in the Results section (“As for the general population, most individuals had an academic degree (47.5%) and worked on-site (53.2%).”) with additional information that was collected in the sociodemographic questionnaire.*

In Measures, the authors of the scales “ODSIS”, “OASIS”, “IES-6” and “The Fear of COVID-19 Scale” should be specified in the text.

- *The authors of the scales were identified in the description of each one of them (please check subsection Measures, section “Methods”).*

The data analysis is in line with the research goals. Nevertheless, the analysis of the variable Risk group for COVID-19, that was ignored in the results, could provide interesting findings.

- *Thank you for your note. As you mention, being part of the risk group may have a strong effect on mental health outcomes. Thus, the importance of considering this factor when interpreting the results of the longitudinal impact of the pandemic was further detailed in the revised version of the manuscript (“Having underlying medical conditions, living in poor housing settings, or integrating a social minority represented a higher risk for those who got sick from COVID-19, making them more vulnerable to develop acute infection and clinical levels of psychological symptoms [65]. Therefore, the longitudinal effects of the study should be interpreted with caution within the context of the pandemic.”).*

The discussion of the results is appropriate and based on recent studies. The authors report the limitations of the study and its implications.

- *Thank you for recognizing our efforts in contributing with an informative and honest submission.*
